# Perspective: Radiotherapy and Body Composition: Unmet Needs in Low- and Middle-Income Countries^☆^

**DOI:** 10.1016/j.advnut.2025.100563

**Published:** 2025-11-14

**Authors:** Alexia J Murphy-Alford, Aaron J Grossberg, Vickie E Baracos, Maha Barbar, Judy Bauer, Jonathan P Bennett, Elena Fidarova, Clifton D Fuller, Anastassia Löser, Amy C Moreno, Anurima Patra, Deepa Puttaswamy, David I Rosenthal, Judy Schoeman, Yavuz Anacak

**Affiliations:** 1 Department of Nuclear Sciences and Applications, Nutritional and Health-Related Environmental Studies Section, International Atomic Energy Agency, Vienna, Austria; 2 Cancer Early Detection Advanced Research Center (CEDAR), Knight Cancer Institute, Oregon Health and Science University, Portland, OR, United States; 3 Brenden-Colson Center for Pancreatic Care, Oregon Health and Science University, Portland, OR, United States; 4 Department of Radiation Medicine, Oregon Health and Science University, Portland, OR, United States; 5 Department of Oncology, Cross Cancer Institute, Edmonton, AB, Canada; 6 Division of Gastroenterology and Nutrition, Department of Pediatrics, King Hussein Cancer Center, Amman, Jordan; 7 Department of Nutrition, Dietetics and Food, Faculty of Medicine, Nursing and Health Science, Monash University, Melbourne, Australia; 8 Department of Epidemiology, University of Hawai’i Cancer Center, Honolulu, HI, United States; 9 Department of Nuclear Sciences and Applications, Applied Radiation Biology and Radiotherapy Section, International Atomic Energy Agency, Vienna, Austria; 10 Department of Radiation Oncology, The University of Texas MD Anderson Cancer Center, Houston, TX, United States; 11 Department of Radiotherapy, University Medical Center Schleswig-Holstein/Lübeck, Lübeck, Germany; 12 Department of Radiology, Tata Medical Center, Kolkata, West Bengal, India; 13 Division of Nutrition, St. John’s Research Institute, St. John’s National Academy of Health Sciences, Bengaluru, Karnataka, India; 14 Division of Paediatric Oncology and Haematology, Department of Paediatrics and Child Health, Faculty of Medicine and Health Sciences, University of Pretoria, Pretoria, South Africa; 15 Department of Paediatrics and Child Health, Faculty of Medicine and Health Sciences, Tygerberg Hospital, Stellenbosch University, Cape Town, South Africa; 16 Department of Radiation Oncology, Ege University Faculty of Medicine, Izmir, Turkey

**Keywords:** body composition, cancer, radiotherapy, low- and middle-income countries, nutrition

## Abstract

Radiotherapy plays a vital role in cancer treatment, yet its effects on patients’ nutritional status can precipitate muscle loss, with significant implications for treatment tolerance and outcomes. Evidence from high-income countries increasingly links radiation-induced muscle loss to adverse clinical outcomes. In contrast, low- and middle-income countries face a stark evidence gap, despite the heightened vulnerability of cancer patients in these settings due to delayed diagnosis, limited access to care, and high rates of co-morbidities. This paper highlights the critical gaps in nutritional care for radiotherapy patients in low- and middle-income countries.

**Statements of Significance**

Radiotherapy can affect body composition and consequently clinical outcomes, yet evidence from low- and middle-income countries remains limited despite heightened risks from delayed care and comorbidities. This perspective argues that generating context-specific body composition data and building evidence for integrating nutrition into radiotherapy services are critical steps to optimize treatment and improve survivorship in resource-limited settings.

## Introduction

Cancer remains a critical global health challenge, representing a leading cause of premature mortality. In 2022, an estimated 20 million new cancer cases and nearly 10 million cancer-related deaths were reported, accounting for ∼17% of all deaths worldwide [1]. The burden of cancer is unequally distributed across countries of varying income levels, with significant disparities in incidence and mortality [2]. In low- and middle-income countries (LMICs), late-stage presentation and limited access to timely and effective treatment are factors affecting mortality [3]. Furthermore, site-specific cancer patterns vary by income level, with prostate and colorectal cancers dominating in high-income countries (HICs) due to lifestyle and screening practices, whereas cervix, liver, and gastric cancers are more prevalent and deadly in LMICs, often linked to infectious aetiologies and inadequate preventive infrastructure [2,4]. These disparities underscore the urgent need for further equitable global strategies in cancer prevention, early detection, and treatment.

Radiotherapy (RT), alongside surgery and systemic therapies, constitutes a major component of cancer care. It is recognized as a safe and effective pillar in modern oncologic therapy. RT is 1 of the most cost-effective cancer treatment modalities, despite the need for substantial capital investment in the facilities and equipment [5]. Evidence-based guidelines suggest that more than half of cancer patients receive ≥1 course of RT during their disease course, either alone or in combination with other modalities [6]. There were 10 million new patients needing RT in 2022, and it was estimated that this would reach 16.5 million new patients in 2050, with Asia estimated to have the highest RT demand in 2050 [7]. RT can be employed as a radical therapy, denoting curative intent for early to local-regionally advanced cancers, organ-sparing treatment for early-stage tumors, or to palliate cancer-related symptoms in advanced, recurrent, or metastatic cancer. RT is an essential element of curative treatment of cancers of the breast, prostate, cervix, head and neck, lung, brain, and sarcomas. Despite the potential of RT, combined with surgery and systemic therapies, to cure a growing number of patients, hundreds of thousands in LMICs lack access to RT, with 31% of LMICs having no RT facility [8]. Efforts by individual countries and international initiatives, such as the International Atomic Energy Agency (IAEA) Rays of Hope, are working to close this gap by establishing or strengthening RT capacity in LMICs [9].

Adults with cancer commonly experience malnutrition, with estimates ranging between 25% and 80% across patient populations, stage, and diagnostic criteria [10]. Patients with gastrointestinal, head and neck, and lung cancers experience the highest levels of malnutrition [[11], [12], [13]]. Cancer-associated malnutrition is primarily driven by reduced food intake due to nutrition-impact symptoms arising from local tumor burden, systemic cytokines, or treatment side effects. Though often curative, RT causes an inflammatory response with acute and late toxicities that may compromise patients’ nutritional status [14]. RT commonly induces oral mucositis, xerostomia, dysphagia, dysgeusia, anorexia, and gastrointestinal disturbances such as nausea, vomiting, and diarrhea [[15], [16], [17]], all of which impair dietary intake. In addition, cancer-related metabolic alterations, including systemic inflammation in response to RT [18] or chemo-RT, increase rates of catabolism in muscle and adipose tissue, futile metabolic cycling, and anabolic resistance, further compromise nutrient utilization and contribute to the development and progression of weight loss and tissue–level body composition changes (i.e., skeletal muscle, adipose tissues and organs) [19]. The most common change in body composition experienced by cancer patients is loss of skeletal muscle mass. Sarcopenia refers to the depletion of skeletal muscle mass to levels associated with increased risk of mortality [[20], [21], [22]]. Loss of skeletal muscle mass can occur with or without loss of fat mass [19]. Muscle loss can be detected in patients with a stable body weight [23]. Patients with obesity can also have underlying sarcopenia; sarcopenic obesity is defined as the co-existence of excess adiposity and low muscle mass/function [24]. Loss of muscle and sarcopenia have been consistently associated with adverse outcomes, and the timely recognition and management of these conditions are critical to optimizing clinical results and enhancing patient resilience throughout the treatment trajectory.

## Unmet Clinical and Research Needs

Despite increasing awareness of the clinical importance of cancer-related loss of muscle, notable disparities continue to affect its recognition, evaluation, and management across global healthcare systems. In HICs, emerging advances in diagnostic imaging, multidisciplinary care models, and evidence-based nutritional interventions are gradually enabling more consistent detection and treatment of body composition changes in oncology patients. In contrast, nutritional support is seldom prioritized for patients undergoing RT in LMICs, where access to RT itself is often limited, let alone access to body composition assessment tools, nutritional services, and integrated cancer care infrastructure. To improve clinical outcomes and reduce global inequities, it is essential to identify these gaps systematically and develop actionable, context-sensitive strategies that strengthen care pathways in LMICs (Table 1) [22,[25], [26], [27], [28], [29], [30], [31], [32], [33], [34], [35], [36], [37], [38], [39], [40], [41], [42], [43], [44], [45], [46], [47], [48], [49], [50], [51], [52], [53], [54], [55]].

**TABLE 1:** Evidence and action framework

Domain	Unmet need	Current evidence from HIC	Implications for LMIC	Recommended action
Impact of RT	Poor understanding of how RT impacts muscle mass in the short and long term	Nutritional status and muscle mass deteriorate throughout RT, even with active nutritional support [[25], [26], [27]]	Limited nutrition management is considered for RT patients	Investigate longitudinal body composition changes during and post-RT in LMIC populations
Outcomes	Limited data on the prognostic impact of RT-related muscle mass changes	Sarcopenia is associated with poor treatment compliance and survival [28,29,[39], [40], [41], [42], [43], [44], [45], [46]] Sarcopenia is associated with RT delays [[28], [29], [30], [31], [32]]. Sarcopenia is associated with poor QoL [[33], [34], [35], [36], [37], [38]]	Limited risk stratification and outcome prediction in LMIC cancer populations Missed preventative opportunities	Conduct prospective studies on body composition changes during RT in LMIC cohorts, including functional, clinical, patient-reported, and survival outcomes
Assessment methods	Lack of access to feasible body composition tools	Body composition methods (BIA, DXA, CT) are valid and feasible in cancer patients [[47], [48], [49], [50], [51],^55^]	Limited implementation of body composition measurements in clinical settings	Validate and implement affordable tools, such as BIA Explore and refine the use of diagnostic CT scans Maximize the use of DXA for body composition as well as bone density
Nutrition integration	Poor integration of nutrition care into oncology workflows	Nutritional management is feasible and effective in cancer patients receiving RT [52]	Lack of systematic support Increased nutritional concerns and outcomes	Develop evidence-based nutrition guidelines tailored for LMICs Embed nutrition assessment and management into RT workflow
Health systems/policy	Limited awareness of the economic value of nutrition support	Limited studies have quantified the cost-effectiveness of nutritional support [22,53,54]	Poor funding and inadequate prioritization of nutrition resources in cancer management	Conduct cost-effectiveness research Use research as evidence to advocate for nutrition management during and beyond cancer treatment

Abbreviations: BIA, bioelectrical impedance analysis; CT, computed tomography; DXA, dual-energy x-ray absorptiometry; HIC, high-income countries; LMIC, low- and middle-income countries; QoL, quality of life; RT, radiotherapy.

### Impact of radiotherapy on body composition

The prevalence of sarcopenia and sarcopenic obesity has been complicated by the definition and assessment criteria used. The reported prevalence of these conditions in patients with cancer receiving RT also ranges widely due to disease stage, patient age, and cancer type. A recent meta-analysis of sarcopenia in solid tumor patients found an overall prevalence of 35%, with rates exceeding 50% in esophageal, prostate, sarcoma, and thyroid cancers; ranging from 35% to 50% in head and neck squamous cell carcinomas, pancreatic, lung, renal, and ovarian cancers; and below 35% in colorectal, gastric, hepatocellular, and breast cancers [56]. The prevalence of sarcopenic obesity in cancer patients has been reported from 1% to 29% [57], with a meta-analysis showing a pooled prevalence of 20% [58], though this was not only in patients receiving RT. Several factors have been identified as significant predictors of sarcopenia, including low BMI (in kg/m^2^), older age, sex, advanced disease stage, smoking history, diabetes mellitus, large tumor size (>4 cm), and lymph node metastases [59].

Despite growing recognition of the presence of sarcopenia as a prognostic factor, the kinetics and trajectory of body composition changes during RT remain inadequately characterized. Studies in head and neck cancer populations suggest that nutritional status and skeletal muscle mass often deteriorate throughout RT, even with active nutritional support [[25], [26], [27]]. Ahern et al. [60] observed dynamic fluctuations in muscle mass during treatment, with some patients developing sarcopenia and others showing signs of reversal. These observations underscore unresolved questions regarding the reversibility of RT-associated sarcopenia and whether recovery confers measurable clinical benefit.

Currently, most data on the effects of RT on cancer comes from HICs. Comparative data in HICs suggest that sarcopenia prevalence is higher in Europe (45.6%) and North America (41.2%) than in Asia (29.6%) [61]. However, data from LMICs remain sparse, contributing to a significant evidence gap in regions where disparities in cancer care infrastructure may exacerbate nutritional challenges. A literature search conducted on the terms for “cancer” AND [“computed tomography” (CT)] AND (“sarcopenia” OR "skeletal muscle index"(SMI) OR “body composition”) reveals over 2500 published articles, of which 92% describe populations in HIC. Inclusive, longitudinal, multicentric, and context-specific research is essential to characterize the impact of RT on body composition to inform the development of targeted interventions.

### Clinical outcomes and prognostic relevance

Sarcopenia has emerged as a significant prognostic factor in oncology, yet its clinical implications in the context of RT remain underexplored, particularly in LMICs. In HIC, sarcopenia has been independently associated with treatment delays [[28], [29], [30], [31], [32]] and reduced quality of life [[33], [34], [35], [36], [37], [38]] among patients receiving RT. Sarcopenic obesity has been linked to higher rates of postoperative complications, prolonged recovery, and increased length of hospital stay [57,58]. The presence of sarcopenia has been associated with poor overall survival in cancer patients, particularly in head and neck [29,[39], [40], [41], [42], [43],^62^], rectal [30,44,45], and lung cancers [46,63]. Despite this growing evidence, a recent pan-cancer review of patients undergoing RT found that only 12 of 26 studies identified sarcopenia as an independent prognostic factor for survival outcomes [64]. These mixed results likely reflect methodological inconsistencies, small sample sizes, and variability in how sarcopenia is defined, highlighting a critical knowledge gap in RT and nutrition research. These limitations hinder the development of robust risk stratification models and predictive tools. The applicability of outcome research from HICs to LMICs is complicated by differences in anthropometric, nutritional, and clinical contexts. For instance, the relationship between BMI and adiposity, and by extension, muscle mass, varies not only between sexes but also between individuals of the same sex across racial and ethnic groups [[65], [66], [67], [68]]. As a result, definitions of abnormally low muscle mass must be tailored to the normal ranges for a given sex, race, and ethnocultural context. In addition, the relevance and weighting of prognostic factors are shaped by access to medical care, health literacy, oncologic practice standards, and technological resources, which vary considerably across populations and regions [[69], [70], [71]]. Consequently, the effect of muscle loss on cancer outcomes in LMICs is likely to differ substantially from that observed in HICs. The absence of prospective, longitudinal data capturing functional, clinical, patient-reported, and survival outcomes in LMIC populations represents a missed opportunity to identify high-risk patients and implement timely, targeted, and context-specific interventions. Addressing this gap is essential for improving treatment outcomes and quality of life for patients undergoing RT in LMICs.

### Assessment methods and feasibility

Accurate and accessible assessment of body composition is essential for identifying sarcopenia and guiding timely nutritional and therapeutic interventions in patients undergoing RT. Unfortunately, these assessments are not standard of care, and many may have availability challenges. CT-based analysis, leveraging existing diagnostic imaging, is gaining traction as a valid, non-invasive, and opportunistic method for sarcopenia detection in cancer patients [72]. This approach has been used in several thousand publications from around the world, and is applicable to all forms of CT studies, the CT component of positron emission tomography-CT (PET-CT) studies, as well as RT planning CT. There is a volume of evidence already acquired using this CT-based approach in patients with cancer, with 222 papers published in the last 5 y; most of these are primary-tumor and treatment-specific and include substantial numbers of patients. For example, a meta-analysis of CT-defined sarcopenia in head and neck cancer included 63 studies comprising data from 14,804 patients [73]. Despite the potential to extract data on muscle and adipose tissue at every instance of cancer imaging, there remains a need to standardize imaging protocols, enhance and validate automated segmentation tools, refine clinical thresholds defining risk of mortality and treatment toxicity, and explore integration prospectively into clinical workflows.

Where CT is not available, other approaches for measuring body composition may be considered, each with advantages and limitations. Anthropometric-based measurements, including BMI, mid-upper arm circumference, and calf circumference, are suited to resource-limited LMIC settings and offer practical and prognostic screening tools [[74], [75], [76], [77], [78]] but lack the specificity required to detect compartmental changes in fat, muscle, and fluid that are critical for precision oncology. Bioelectrical impedance analysis presents a promising non-ionizing option for routine monitoring owing to its portability and low cost [[79], [80], [81]], with phase angle, a marker of cellular integrity and surrogate for muscle quality, being increasingly recognized as a valuable prognostic indicator in oncology [[47], [48], [49], [50],^82^]. Dual-energy x-ray absorptiometry has been shown to be a valuable body composition technique in cancer patients [51] but is more commonly utilized for bone mineral density and diagnosis of osteoporosis. Total appendicular lean soft tissue is used in clinical and research settings as a surrogate for muscle mass [83] and has been associated with early mortality in cancer patients [84]. However, dual-energy x-ray absorptiometry remains underutilized beyond bone mineral density assessments in the oncology setting due to limited access and lack of clinical familiarity.

A major limitation in applying body composition techniques to diagnose sarcopenia in LMICs is the reliance on reference populations derived primarily from HICs [85], despite substantial variation in normative body composition across populations [65‒68]. Consequently, diagnostic thresholds must be context-specific. Therefore, there is an urgent need for studies that establish clinically relevant cut-offs tailored to LMIC settings and assess their feasibility and effectiveness in routine care.

Despite its clinical relevance, body composition assessment is infrequently incorporated into routine cancer care in HICs, and even less so in LMICs, due to limitations in time, cost, infrastructure, and availability of trained personnel. There is an urgent need for research to validate and refine body composition techniques for use in cancer patients, so that affordable, quick, and scalable body composition assessment tools can be implemented within standard oncology care pathways across both HIC and LMICs. Target areas should include leveraging routinely acquired diagnostic CT scans and expanding access to alternative body composition approaches that are adapted to resource-limited settings and amenable to routine clinical workflows.

### Integration of nutrition into oncology workflows

In HICs, studies have demonstrated that nutritional interventions during RT are both feasible and effective, leading to improved treatment adherence, reduced toxicity, and better quality of life [52]. However, in LMICs, systematic nutrition support is often lacking due to limited resources, insufficient clinical infrastructure, and a lack of trained personnel. This gap is especially concerning given the increased nutritional vulnerability of patients undergoing RT, who are at high risk of developing or worsening malnutrition and sarcopenia. Embedding routine nutrition assessment into RT care pathways is essential to ensure timely identification of at-risk patients and to deliver appropriate interventions. Furthermore, there is a need to develop evidence-based nutrition guidelines tailored to the specific challenges and local dietary contexts of LMICs. Strengthening the integration of body composition assessment-informed nutrition management into oncology care will require coordinated efforts across clinical practice, education, and policy.

### Health systems and policy considerations

At the health systems and policy level, there is limited recognition of the economic value of integrating nutrition support into cancer care, particularly in LMICs. In HICs, emerging evidence suggests that nutrition interventions are not only clinically beneficial but also cost-effective, contributing to reduced treatment-related complications, shorter hospital stays, and lower overall healthcare costs [22,53,54]. However, in LMICs, nutrition services are often underfunded and deprioritized within oncology programs, resulting in inadequate access to trained personnel, infrastructure, and therapeutic nutrition products. This lack of investment undermines the potential for nutrition to serve as a low-cost, high-impact intervention in cancer care. To address this, there is a pressing need for context-specific cost-effectiveness research that quantifies the clinical and economic benefits of nutrition support for cancer patients receiving RT in LMICs. Such evidence can be leveraged to inform policy development, advocate for resource allocation, and embed nutrition as a standard component of comprehensive cancer care.

In conclusion, robust evidence from HICs confirms that cancer and cancer treatments exert a significant impact on muscle mass with a high reported incidence of sarcopenia, with downstream effects on treatment tolerance, clinical outcomes, and survivorship. However, in LMICs, data remain limited, despite the presence of additional confounding factors such as delayed diagnosis and treatment, limited healthcare infrastructure, and high burden of co-morbidities that may intensify body composition changes and their consequences. To promote sustainable integration of nutritional care into RT services in LMICs, it is essential to highlight its established clinical and economic value. Generating rigorous, context-specific evidence will be key to informing policy decisions, allocating resources, and embedding nutrition into routine RT care. Encouragingly, technologies such as the use of clinically indicated CT scans offer feasible pathways for body composition assessment beyond BMI in patients undergoing RT. Facilitating their adoption can help close critical evidence gaps, elucidate the impact of RT on muscle mass, and improve prognostic evaluation through both clinical and patient-reported outcomes. Ultimately, strengthening nutritional support capacity alongside RT services represents a dual strategy to optimize cancer care and enhance outcomes for patients in LMICs.

## Author contributions

The authors’ responsibilities were as follows– AJM-A, AJG, VEB, MB, JB, JPB, CDF, AL, ACM, AP, DP, DIR, JS, YA: designed the review; AJM-A, AJG, VEB, MB, JB, JPB, CDF, AL, ACM, AP, DP, DIR, JS, YA: wrote the manuscript; AJM-A: had primary responsibility for final content; and all authors: read and approved the final manuscript.

## Funding

The authors reported no funding received for this study.

## Conflict of interest

The authors report no conflicts of interest.
